# Supplementary material for: MetaRibo-Seq measures translation in microbiomes
Source: Nat Commun. 2020 Jun 29;11:3268. doi: 10.1038/s41467-020-17081-z (PMC7324362; doi:10.1038/s41467-020-17081-z)
Supplement: Supplementary file 10 — Supplementary Data 7 [file 41467_2020_17081_MOESM10_ESM.zip › File2/Confidence_High_Taxonomy/5953_out.krona.html]

Javascript must be enabled to view this page.

members
magnitude
magnitudeUnassigned
count
unassigned
taxon
rank

5953\_out

14

14
2
superkingdom


SRS019267\_contig\_number\_contig-100\_22009.22009SRS023715\_contig\_number\_contig-100\_17198.106932SRS077392\_contig\_number\_contig-100\_20993.20994SRS077552\_contig\_number\_contig-100\_13226.54836SRS1041037\_contig\_number\_contig-100\_8020.8021SRS143342\_contig\_number\_5514
6
phylum
14
1239

186801
8
class

order
8
186802

8
186803
family

genus
1506577
8


SRS015663\_contig\_number\_16725SRS024132\_contig\_number\_39957SRS074964\_contig\_number\_465SRS077194\_contig\_number\_contig-100\_1243.1243SRS104036\_contig\_number\_contig-100\_8354.8355SRS142503\_contig\_number\_12781SRS146764\_contig\_number\_26967SRS149181\_contig\_number\_contig-100\_13190.55018
species
2053632
8
